# The influence of the value of children on the fertility intentions of people of childbearing age in China

**DOI:** 10.3389/fsoc.2025.1686244

**Published:** 2025-11-24

**Authors:** Jinming Fang, Ling Liu

**Affiliations:** 1 Office of Quality Management, Wuhan Fourth Hospital, Wuhan, China; 2 Management Office of Clinical Research Center, The Central Hospital of Wuhan, Tongji Medical College, Huazhong University of Science and Technology, Wuhan, China

**Keywords:** fertility intention, value of children, psychological affection, familial responsibility, CFPS

## Abstract

**Objective:**

This study aims to explore the mechanisms through which the multidimensional reconstruction of childbearing values in the post-pandemic era influences fertility intentions among Chinese individuals of reproductive age, while elucidating the interplay between economic rationality and cultural norms in fertility decision-making, thereby providing theoretical foundations for targeted fertility policy formulation.

**Methods:**

Utilizing data from the 2022 China Family Panel Studies (CFPS), we analyzed a sample of 1,758 individuals of reproductive age. Factor analysis was employed to extract three dimensions of childbearing values—psychological affection, economic utility, and familial responsibility. A logistic regression model incorporating control variables (gender, household registration, education, etc.) was constructed, followed by heterogeneity analyses across demographic subgroups.

**Results:**

All three dimensions of childbearing values exhibited significant positive effects on fertility intentions, with familial responsibility demonstrating the strongest impact (coefficient = 0.249, *p* < 0.01). Heterogeneity analysis revealed that psychological affection predominantly influenced females (coefficient = 0.316, *p* < 0.05) and rural populations, while economic utility exerted a pronounced effect on high-income groups (coefficient = 0.306, *p* < 0.05). Educational attainment consistently enhanced fertility intentions (coefficient = 0.206, *p* < 0.01).

**Conclusion:**

Fertility decisions emerge as a dynamic interplay between cultural values and resource endowments. Sustainable enhancement of fertility intentions necessitates differentiated policy interventions that reinforce familial responsibility, alleviate economic constraints, and address emotional needs.

## 1 Background

China's demographic landscape has undergone profound transformations in recent decades. The mortality rate has stabilized at approximately 7‰, while the birth rate has exhibited a persistent downward trajectory, declining from 14.03‰ in 2000 to 6.39‰ in 2023 [National Bureau of Statistics of China (NBoSo), 2024]. This demographic shift has resulted in a negative natural population growth rate of −1.48‰ [National Health Commission of China (NHC), 2023], compounded by a total fertility rate decrease from 1.22 in 2000 to 1.09 in 2022 (According to data from the National Bureau of Statistics of China)—significantly below the 2.1 replacement level necessary for population stability.

**Figure 1:**
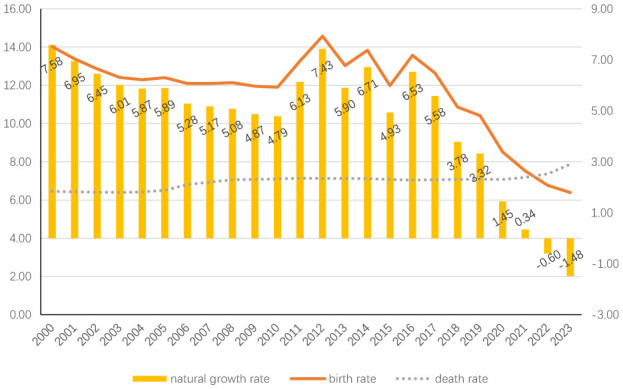
Birth rate, mortality rate and natural growth rate of China population from 2000 to 2023 (unit: ‰).

**Figure 2:**
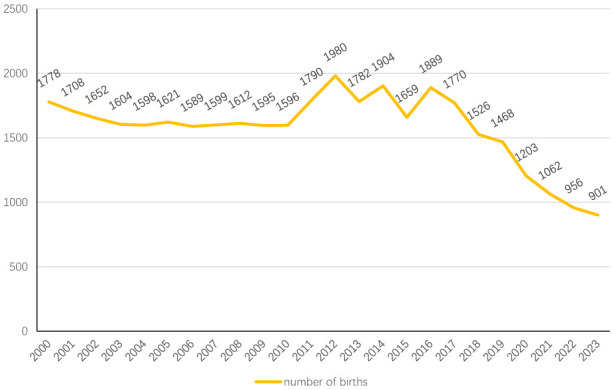
Number of births in China from 2000 to 2023 (unit: 10,000).

To address ultra-low fertility challenges, Chinese policymakers have progressively enacted reproductive policy adjustments: the (Chen et al., 2023; Yue et al., 2023). Complementary measures including childcare subsidies and enhanced parental leave provisions have been concurrently implemented (Chen et al., 2023). Despite these interventions, demographic projections remain unmet. Live births plummeted to 9.01 million in 2023, marking a 54.5% decline from the 2012 peak of 19.80 million. Empirical evidence reveals limited reproductive enthusiasm among people of childbearing age: only 10.4% of two-child mothers demonstrated third-child intentions in controlled cohort studies (Yang et al., 2024), while post-policy national surveys indicate merely 9.6% of two-child households planned additional births (Zhao et al., 2024). These persistent fertility resistance patterns underscore complex socioeconomic determinants (see Figures 1, 2 for details).

The persistent decline in fertility rates has positioned fertility intention research as a critical focus in population policy development. Previous studies have systematically investigated the associations between reproductive decisions and demographic characteristics (age, gender, health status, marital status) (Ren et al., 2024), household economic conditions (Yang et al., 2024), and administrative policy interventions.

At the individual level, males demonstrate significantly higher fertility intentions than females (Jing et al., 2022), with spousal consensus in reproductive motivation emerging as a pivotal determinant (Guo et al., 2022). Educational attainment exhibits an inverse relationship with fertility aspirations (Xiang et al., 2023), while the dual mediating effects of anxiety and subjective wellbeing substantially suppress reproductive motivation (Yang et al., 2023). A review on the fertility intention of people living with HIV indicates that in China, childlessness exerts a more significant impact on fertility intention. In contrast, in developing and developed countries, higher educational attainment exerts a weaker impact on fertility intention, and sometimes even a negative one (Lin et al., 2024).

Household-level analyses identify economic constraints—particularly housing costs and childcare expenditures—as primary inhibitory factors (Damtew et al., 2025). Empirical evidence from Shanghai indicates that families allocating over 30% of monthly income to childcare experience significant reductions in second-child intentions (Ning et al., 2022). Negative economic expectations further diminish fertility propensity (Matera et al., 2022), a pattern corroborated by South Korean studies demonstrating inverse correlations between household income and fertility intentions (Zhuang et al., 2025). Chinese married cohorts exhibit reduced reproductive intentions due to heightened health risk perception and diminished social support networks (Ning et al., 2024), while high-income families display suppressed fertility desires associated with the high time cost (Peng et al., 2023). Family structure exerts bidirectional influences: individuals raised in multi-child families develop stronger fertility intentions through sibling care experiences, whereas current family size expansion inversely correlates with reproductive decisions (Fantaye et al., 2025).

Macro-level investigations highlight the compound effects of sociocultural norms and policy frameworks on fertility behaviors. Public childcare systems, flexible work arrangements (Xiong et al., 2022), and reproductive healthcare policies (Xu et al., 2024) demonstrate positive motivational effects, though empirical studies emphasize that only integrated implementation of financial subsidies, parental leave schemes, and childcare services achieves measurable fertility improvements (Guetto et al., 2025). Cross-national comparisons reveal cultural moderation effects: German studies document post-pregnancy loss intensification of fertility intentions (Beringer and Milewski, 2024), Finnish research identifies lower ideal family size among single-parent households (Karhunen et al., 2023), while Iranian interventions report 20% fertility intention increases in single-child families following educational programs (Zhu et al., 2022). A Chinese study shows that distrust of local governments can negatively affect the willingness to have children (Zheng et al., 2024).

The value of children (VOC) serves as a core psychological and socioeconomic determinant of fertility intentions, with its impact shaped by both positive utility perception and negative cost evaluation. The positive dimension of VOC (VOC+) encompasses multifaceted values. Psychologically, “primary group ties and affiliation” as well as emotional companionship are identified as key drivers, especially in urban populations where psychological value outweighs instrumental functions. Socioeconomically, economic benefits and “morality” dimensions correlate significantly with ideal fertility, while intergenerational welfare concerns—rooted in parental altruism—also enhance willingness to bear children (Guo et al., 2023). Conversely, the cost dimension (VOC-) exerts inhibitory effects. Recognition of “responsibility for education” and “loss of personal freedom” reduces ideal family size, with women and urban residents more sensitive to external and psychological burdens. Notably, the impact exhibits pronounced heterogeneity. Rural groups prioritize instrumental value, which positively predicts fertility intentions (Fu et al., 2023), whereas urban populations' decisions hinge on psychological value and cost trade-offs. Additionally, education-driven shifts from “child-centered” to self-focused values weaken VOC's positive influence, particularly among middle-income groups facing intense educational investment competition.

The COVID-19 pandemic, as an external shock, has further exacerbated the low fertility challenge that China was already facing. On the one hand, the uncertainty about the future and economic pressures brought about by the COVID-19 pandemic have significantly reduced people's fertility intention. On the other hand, due to the reduced accessibility of medical services during the pandemic, many couples planning to conceive have adjusted their reproductive plans (Chen et al., 2022). In the post-pandemic era, how to rebuild social confidence and promote childbearing and childrearing has become a key issue concerning the country's long-term development.

Based on the China Family Panel Studies in 2022, this study focuses on analyzing the impact of the multi—dimensional reconstruction of the value of children in the post—pandemic era on fertility intention. By employing hierarchical regression models to identify differences in the perception of the value of children among different generations, urban and rural areas, and income groups, it provides an empirical basis for the design of targeted fertility policies. This study holds significant practical relevance and academic frontier value.

## 2 Research design

### 2.1 Data sources

The data used in this study are sourced from the China Health Statistics Yearbook and the China Family Panel Studies (CFPS). The China Family Panel Studies (CFPS) is a longitudinal survey targeting communities, families, and individuals. It covers 25 provinces in China and is nationally representative. The CFPS has been conducted since 2010, with surveys carried out every 2 years. The CFPS provides rich information regarding economic aspects, demographic characteristics, and family-level details, such as education, family dynamics, employment, health, and numerous other economic factors. The CFPS data are nationally representative longitudinal data with a sample of sufficient size. The data used in this paper are from the CFPS in 2022.

According to the requirements of this research, based on the individual questionnaires, this study matched the family member questionnaires and the family economic questionnaire database. It retained the population within the childbearing age range (that is, it retained men aged 22–60 and women aged 20–49). Among the variables, fertility intention, the value of children, and relevant control variables were retained. After excluding the missing values of the variables, a total of 1,758 samples were obtained.

### 2.2 Variable definitions

#### 2.2.1 Explained variable

In this paper, the “Fertility Intention in the Next Two Years” in the CFPS (2022) questionnaire (coded as qka205) is used as a measurement indicator of fertility intention, and a binary classification assignment is carried out. Zero represents no fertility intention, and 1 represents having a fertility intention.

#### 2.2.2 Core independent variable

This paper uses the scale of the value of children as the core independent variable. This scale consists of a total of 9 questions (coded as qme201 – qme209), asking the respondents, “The following lists the general reasons why people want to have children. Please tell me your opinions based on your own personal experiences.” The responses of people outside the childbearing age range are almost all missing values, leading to a decrease in the sample size. The specific contents of the 9 questions are shown in Table 1.

**Table 1:** Descriptive statistics.

**Variable**	**Variable definition**	**Obs**	**Mean**	**Std. Dev**.	**Min**	**Max**
qka205(fertility intention)	Whether will have a child within the next 2 years, 0 = No, 1 = Yes	1,758	0.169	0.375	0	1
qme201	Having children is to get help when you are old. 1 = Strongly Disagree, 2 = Disagree, 3 = Neutral, 4 = Agree, 5 = Strongly Agree	1,758	2.892	0.75	1	5
qme202	Having children is to continue the family lineage. 1 = Strongly Disagree, 2 = Disagree, 3 = Neutral, 4 = Agree, 5 = Strongly Agree	1,758	2.728	0.765	1	5
qme203	Having children is to get economic help for your family. 1 = Strongly Disagree, 2 = Disagree, 3 = Neutral, 4 = Agree, 5 = Strongly Agree	1,758	2.42	0.789	1	5
qme204	Having children is for the joy of watching them grow up. 1 = Strongly Disagree, 2 = Disagree, 3 = Neutral, 4 = Agree, 5 = Strongly Agree	1,758	3.234	0.563	1	5
qme205	Having children is for the happiness of having them around. 1 = Strongly Disagree, 2 = Disagree, 3 = Neutral, 4 = Agree, 5 = Strongly Agree	1,758	3.239	0.566	1	5
qme206	Having children is for the joy of having a baby. 1 = Strongly Disagree, 2 = Disagree, 3 = Neutral, 4 = Agree, 5 = Strongly Agree	1,758	3.219	0.565	1	5
qme207	Having children is to make the family more important in your life. 1 = Strongly Disagree, 2 = Disagree, 3 = Neutral, 4 = Agree, 5 = Strongly Agree	1,758	3.108	0.634	1	5
qme208	Having children is to enhance your sense of responsibility. 1 = Strongly Disagree, 2 = Disagree, 3 = Neutral, 4 = Agree, 5 = Strongly Agree	1,758	3.12	0.567	1	5
qme209	Having children is to strengthen family ties. 1 = Strongly Disagree, 2 = Disagree, 3 = Neutral, 4 = Agree, 5 = Strongly Agree	1,758	2.982	0.639	1	5
gender	Gender, 0 = Female, 1 = Male	1,758	0.478	0.5	0	1
age	Age	1,758	35.89	7.08	22	49
hukou	household registration, 1 = Agricultural, 0 = Non-agricultural	1,758	0.821	0.383	0	1
spouse	Marital status: 0 = No spouse, 1 = Has spouse	1,758	1	0	1	1
medsure_dum	Whether purchased medical insurance, 0 = No, 1 = Yes	1,758	0.923	0.267	0	1
pensioninsurance_dum	Whether participated in pension insurance: 0 = No, 1 = Yes	1,758	0.647	0.478	0	1
edu	Education level: 0 = Illiterate/Semi-illiterate, 1 = Primary school, 2 = Junior high school, 3 = Senior high school, 4 = College and above	1,758	2.392	1.206	0	4
jiankang	Health status 1 = “Unhealthy” 2 = “Average” 3 = “Healthy”	1,758	2.812	0.538	1	3
ln_fincome1_per	Natural logarithm of per capita family income (yuan)	1,758	10.03	0.89	7.523	12.525
familysize22	Family size	1,758	4.761	2.022	1	16
region	Region: 1 = East, 2 = Central, 3 = West	1,758	1.893	0.824	1	3

#### 2.2.3 Control variables

The control variables mainly include demographic characteristic variables, family characteristic variables, and regional variables. The specific demographic characteristics are as follows: age (age), household registration (hukou), marital status (spouse; since all the people who can answer the fertility intention question are married, this variable will be excluded in the subsequent analysis), education level (edu), health status (jiankang), medical insurance (medsure_dum), and endowment insurance (pensioninsurance_dum). The family characteristics include the logarithm of the per capita annual income of the family (ln_fincome1_per) and the number of family members (familysize22). The regional variable (region) is generated based on provincial variables and is divided into the eastern region, the central region, and the western region.

## 3 Results

### 3.1 Statistical description

Through descriptive statistics, it can be found that 16.9% of the samples indicated that they would have children within the next 2 years. The range of people of childbearing age who answered questions about fertility wishes was between 22 and 49 years old. The overall health status of the population was relatively high. For detailed information, please refer to Table 1.

### 3.2 Factor analysis

For the scale of the value of children, after recoding, a correlation analysis and a Kaiser-Meyer-Olkin (KMO) test were first conducted. The correlation analysis showed that there were statistical significances among the 9 questions (see Table 2). The KMO test resulted in a KMO value of 0.837 and a *p*-value less than 0.001, indicating that it was suitable for factor analysis.

**Table 2:** Correlation analysis results.

**Variable**	**qme201_new**	**qme202_new**	**qme203_new**	**qme204_new**	**qme205_new**	**qme206_new**	**qme207_new**	**qme208_new**	**qme209_new**
qme201_new	1								
qme202_new	0.520^***^	1							
qme203_new	0.469^***^	0.542^***^	1						
qme204_new	0.096^***^	0.139^***^	0.142^***^	1					
qme205_new	0.153^***^	0.156^***^	0.155^***^	0.544^***^	1				
qme206_new	0.167^***^	0.193^***^	0.178^***^	0.542^***^	0.608^***^	1			
qme207_new	0.268^***^	0.311^***^	0.278^***^	0.305^***^	0.374^***^	0.412^***^	1		
qme208_new	0.248^***^	0.285^***^	0.268^***^	0.311^***^	0.377^***^	0.409^***^	0.528^***^	1	
qme209_new	0.272^***^	0.305^***^	0.315^***^	0.277^***^	0.335^***^	0.371^***^	0.464^***^	0.536^***^	1

^***^p < 0.01.

Subsequently, factor analysis was carried out. According to the scree plot, three common factors were selected for extraction. Through rotation using the varimax method, it was found that the cumulative contribution rate of the three common factors reached 68.8%. The questions corresponding to the common factors are shown in Table 3. Based on the meanings of the questions, they were respectively named as factor1: “Psychological and Emotional Value”, factor2: “Economic Utility Value”, and factor3: “Family Responsibility Value”.

**Table 3:** Factor analysis results.

**Variable**	**Factor1**	**Factor2**	**Factor3**	**Uniqueness**
qme201_new		0.7946		0.3472
qme202_new		0.8161		0.2948
qme203_new		0.7929		0.3369
qme204_new	0.8354			0.288
qme205_new	0.8174			0.2762
qme206_new	0.7863			0.2866
qme207_new			0.7285	0.365
qme208_new			0.8033	0.2792
qme209_new			0.7682	0.3341

Blanks represent abs(loading) <0.4.

### 3.3 Regression analysis

#### 3.3.1 Basic regression

Since the dependent variable in this study is a binary variable, binary logistic regression was employed to analyze the data. Models 1–3 are regressions of the three common factors on the dependent variable, respectively. Model 4 is a regression conducted after adding the independent variables of personal factors to the three common factors. On the basis of Model 4, Model 5 added the independent variables of family, and Model 6 further added regional variables. The specific results are shown in Table 4. A collinearity test was carried out for all independent variables, and it was found that there was no situation of multicollinearity (see Supplementary Table S1).

**Table 4:** The basic logistic regression model of child value on fertility intention.

**Variable**	**(1)**	**(2)**	**(3)**	**(4)**	**(5)**	**(6)**
	**qka205(fertility intention)**	**qka205(fertility intention)**	**qka205(fertility intention)**	**qka205(fertility intention)**	**qka205(fertility intention)**	**qka205(fertility intention)**
factor1 (Psychological and emotional value)	0.208^***^ (0.070)			0.168^**^ (0.078)	0.177^**^ (0.079)	0.178^**^ (0.080)
factor2 (Economic utility value)		−0.091 (0.064)		0.143^*^ (0.076)	0.222^***^ (0.080)	0.221^***^ (0.080)
factor3 (Family responsibility value)			0.154^**^ (0.070)	0.200^***^ (0.075)	0.250^***^ (0.077)	0.249^***^ (0.078)
Gender				0.603^***^ (0.147)	0.556^***^ (0.151)	0.555^***^ (0.151)
Age				−0.163^***^ (0.014)	−0.167^***^ (0.014)	−0.167^***^ (0.014)
hukou				0.395^*^ (0.206)	0.603^***^ (0.216)	0.603^***^ (0.216)
edu				0.389^***^ (0.072)	0.204^***^ (0.078)	0.206^***^ (0.079)
jiankang				0.178 (0.170)	0.150 (0.176)	0.152 (0.176)
medsure_dum					−0.084 (0.276)	−0.086 (0.276)
pensioninsurance_dum					0.181 (0.164)	0.180 (0.164)
ln_fincome1_per					0.450^***^ (0.100)	0.458^***^ (0.102)
familysize22					−0.190^***^ (0.044)	−0.191^***^ (0.045)
1. Region						0.000 (0.000)
2. Region						0.047 (0.175)
3. Region						0.076 (0.188)
_cons	−1.622^***^ (0.065)	−1.597^***^ (0.064)	−1.614^***^ (0.065)	1.636^**^ (0.759)	−1.586 (1.325)	−1.714 (1.361)
*N*	1,758	1,758	1,758	1,758	1,758	1,758
Pseudo R^2^	0.006	0.001	0.003	0.189	0.232	0.232
chi^2^	9.266	2.002	5.070	301.909	369.731	369.907

In Models 1–3, the independent variables are the three common factors, respectively. For Models 4–6, on the basis of including the three common factors together, demographic characteristic variables, family characteristic variables, and regional variables were gradually incorporated.

Robust standard error in parentheses; ^***^ *p* < 0.01, ^**^ *p* < 0.05, ^*^ *p* < 0.1.

From the perspective of the regression results, from Model 1 to Model 6, the Pseudo R^2^ of the models increased (from 0.006 to 0.232), indicating that after adding control variables and multiple factors, the explanatory power has significantly enhanced.

Among the core independent variables, Factor 1 (“Psychological and Emotional Value”) always has a significant positive impact on fertility intention (the coefficient in Model 1 is 0.208, and the coefficient in Model 6 is 0.178). This shows that the more one values the emotional satisfaction brought by childbearing, the stronger the fertility intention will be. Factor 2 (“Economic Utility Value”) is not significant in the separate regression (the coefficient in Model 2 is −0.091), but it is significantly positive after adding control variables (the coefficient in Model 6 is 0.221). This means that after controlling factors such as income and education, economic support and old-age support will significantly increase the fertility intention, and it also indicates that its effect needs to be revealed after separating the economic background. Factor 3 (“Family Responsibility Value”) always has a significant positive impact on fertility intention (the coefficient in Model 3 is 0.154, and the coefficient in Model 6 is 0.249). This shows that traditional family responsibility (such as making the family more important and increasing kinship ties) is a stable driving force for fertility intention.

Among the control variables, the fertility intention of men is significantly higher (the coefficient is 0.555), reflecting the differences in gender roles. The older the age, the lower the fertility intention (the coefficient is −0.167), which is in line with the life cycle theory. In terms of household registration, the rural household registration significantly promotes the fertility intention (the coefficient is 0.603), which is related to the household registration welfare and cultural traditions. In terms of education, the higher the education level, the stronger the fertility intention (the coefficient is 0.206, *p* < 0.01). The higher the per capita family income, the stronger the fertility intention (the coefficient is 0.458), reflecting that the economic ability can relieve the pressure of childbearing. The family size inhibits the fertility intention (the coefficient is −0.191), which is in line with the resource dilution theory. The effect of the region is not significant.

The overall situation of the model shows that the fertility intention is the result of the combined effect of psychological values, demographic characteristics, and economic conditions. Among them, the “Family Responsibility Value” (Factor 3) has the greatest impact among the three factors.

#### 3.3.2 Analysis of the situation of Factor 2

As can be seen from the basic regression, Factor 2 is not significant in the separate regression (Model 2), but becomes significant after adding other variables (Models 4-6). Since Factor 2 represents the “Economic Utility Value”, in order to verify whether the independent effect of Factor 2 is masked, we constructed Models 7–10 respectively to examine its effect. Specifically, Model 7 only includes Factor 2 (equivalent to Model 2); Model 8 adds income and education variables; Model 9 adds the family size variable, Factor 1 and Factor 3; and Model 10 adds the interaction term between Factor 2 and economic income. These steps are designed to gradually separate the confounding effects of economic constraints, family structure and value competition on the core effect. The results are shown in Table 5.

**Table 5:** Regression analysis to validate the independent effect of Factor 2.

**Variable**	**(7)**	**(8)**	**(9)**	**(10)**
	**qka205(fertility intention)**	**qka205(fertility intention)**	**qka205(fertility intention)**	**qka205(fertility intention)**
factor2 (Economic utility value)	−0.091 (0.063)	0.141^**^ (0.069)	0.197^***^ (0.069)	1.498^*^ (0.820)
ln_fincome1_per		0.442^***^ (0.085)	0.294^***^ (0.086)	0.293^***^ (0.086)
edu		0.386^***^ (0.065)	0.401^***^ (0.066)	0.405^***^ (0.066)
familysize22			−0.215^***^ (0.048)	−0.218^***^ (0.048)
factor1 (psychological and emotional value)			0.205^***^ (0.070)	0.198^***^ (0.071)
factor3 (family responsibility value)			0.258^***^ (0.071)	0.260^***^ (0.071)
factor2 (economic utility value) ^*^ ln_fincome1_per				−0.125 (0.078)
_cons	−1.597^***^ (0.064)	−7.096^***^ (0.855)	−4.739^***^ (0.952)	−4.750^***^ (0.944)
*N*	1,758	1,758	1,758	1,758
Pseudo *R*^2^	0.001	0.066	0.095	0.097
chi^2^	2.080	81.285	107.898	110.128

Model 7 only includes Factor 2; Model 8 adds income and education variables; Model 9 adds the family size variable, Factor 1 and Factor 3; and Model 10 adds the interaction term between Factor 2 and economic income.

Robust standard error in parentheses; ^***^ p <0.01, ^**^ p <0.05, ^*^ p <0.1.

In Model 7, the coefficient of Factor 2 is negative (−0.0908) and not significant. This indicates that when other variables are not controlled, there is no significant association between the economic utility value and the fertility intention. It is considered that there is a spurious negative correlation caused by the bias of omitted variables. After adding income and education variables in Model 8, the coefficient of Factor 2 turns to be positively significant (0.1412^**^). After taking into account the separation of the economic background, the independent promoting effect of the utilitarian motivation becomes evident. Combined with the fact that income (0.4419^***^) and education (0.3861^***^) significantly promote fertility, the possible reasons include: (1) Economic ability releases the fertility motivation. That is, even if high-income earners have utilitarian motivations, they can afford the costs of childbearing, and thus are more likely to have children. (2) Education strengthens rational choice. That is, highly educated groups are more likely to plan childbearing through systematic evaluation and rational analysis.

In Model 9, after adding the family structure and other common factors, the coefficient of Factor 2 further increases (0.1969^***^). The family size inhibits fertility (−0.2149^***^), while psychological and emotional factors (0.2045^**^) and family responsibility factors (0.2581^***^) significantly promote fertility. This shows that after separating the influences of emotional and responsibility values, the independent effect of the utilitarian motivation is enhanced, indicating that it is a unique driving force for fertility decision-making. In Model 10, after adding the interaction term between Factor 2 and income, the coefficient of the interaction term is −0.125, which is not significant, and the main effect of Factor 2 (1.498^*^) still exists. This suggests that the increase in income may weaken the promoting effect of the utilitarian motivation (high-income earners rely less on their children for old-age support), but there is insufficient statistical support.

#### 3.3.3 Robustness test

To test the robustness of the basic regression, we employed the Probit model and the linear model for estimation. As can be observed in Table 6.

**Table 6:** The robustness test.

**Variable**	**(1)**	**(2)**	**(3)**
	**Logit**	**Probit**	**OLS**
factor1 (psychological and emotional value)	0.178^**^ (0.080)	0.101^**^ (0.044)	0.019^**^ (0.008)
factor2 (economic utility value)	0.221^***^ (0.080)	0.128^***^ (0.044)	0.030^***^ (0.009)
factor3 (family responsibility value)	0.249^***^ (0.078)	0.142^***^ (0.042)	0.028^***^ (0.008)
Gender	0.555^***^ (0.151)	0.294^***^ (0.085)	0.052^***^ (0.017)
Age	−0.167^***^ (0.014)	−0.091^***^ (0.007)	−0.017^***^ (0.001)
hukou	0.603^***^ (0.216)	0.334^***^ (0.115)	0.076^***^ (0.022)
medsure_dum	−0.086 (0.276)	−0.067 (0.160)	−0.006 (0.033)
pensioninsurance_dum	0.180 (0.164)	0.102 (0.091)	0.024 (0.018)
edu	0.206^***^ (0.079)	0.098^**^ (0.044)	0.019^**^ (0.009)
jiankang	0.152 (0.176)	0.066 (0.092)	0.003 (0.012)
ln_fincome1_per	0.458^***^ (0.102)	0.242^***^ (0.056)	0.055^***^ (0.011)
familysize22	−0.191^***^ (0.045)	−0.102^***^ (0.025)	−0.025^***^ (0.004)
1. Region	0.000 (0.000)	0.000 (0.000)	0.000 (0.000)
2. Region	0.047 (0.175)	0.029 (0.096)	0.004 (0.020)
3. Region	0.076 (0.188)	0.058 (0.105)	0.018 (0.021)
_cons	−1.714 (1.361)	−0.801 (0.716)	0.197 (0.137)
*N*	1,758	1,758	1,758
Pseudo *R*^2^	0.232	0.227	
*R* ^2^			0.186
*F*			24.273
chi^2^	369.907	230.613	

The Probit model and the linear model were employed to test the robustness of the basic regression.

Robust standard error in parentheses; ^***^ p <0.01, ^**^p <0.05, ^*^p <0.1.

It can be seen that the promoting effects of the core variables (the three common factors) on fertility intention are significant and in the same direction in all three models, indicating the robustness of the effects. Among the control variables, the effects of gender, age, household registration (hukou), income (ln_fincome1_per), and family size (familysize22) are all significant and in the same direction, demonstrating the robustness of their effects. However, medical insurance (medsure_dum) and endowment insurance (pensioninsurance_dum) are not significant. The significance and direction of both the core variables and the control variables are highly consistent across the three models, which strongly validates the reliability of the conclusions.

#### 3.3.4 Heterogeneity analysis

A further analysis was conducted on the differences in fertility intention in terms of gender, household registration, age, and income. For gender and household registration, binary variables were directly used and incorporated into the model. Since age and income are continuous variables, they were first grouped and then included in the model. Specifically, the age groups were divided into three groups according to gender (for females: 20–29, 30–39, 40–49 years old; for males: 22–34, 35–47, 48–60 years old), and income was divided into three groups according to tertiles. The results are shown in Tables 7, 8.

**Table 7:** Analysis of gender and hukou heterogeneity.

**Variable**	**(1)**	**(2)**	**(3)**	**(4)**
	**Female**	**Male**	**Rural**	**Urban**
factor1 (psychological and emotional value)	0.316^**^ (0.134)	0.077 (0.092)	0.205^**^ (0.085)	0.050 (0.186)
factor2 (economic utility value)	0.255^**^ (0.111)	0.176 (0.114)	0.157^*^ (0.086)	0.463^**^ (0.198)
factor3 (family responsibility value)	0.299^***^ (0.109)	0.201^*^ (0.105)	0.208^**^ (0.084)	0.488^**^ (0.199)
Gender			0.468^***^ (0.165)	1.198^***^ (0.451)
Age	−0.166^***^ (0.020)	−0.171^***^ (0.018)	−0.165^***^(0.015)	−0.186^***^ (0.036)
hukou	0.995^***^ (0.339)	0.249 (0.274)		
medsure_dum	0.218 (0.441)	−0.348 (0.389)	−0.075 (0.322)	0.043 (0.807)
pensioninsurance_dum	0.113 (0.238)	0.225 (0.238)	0.124 (0.178)	0.677 (0.544)
edu	0.188^*^ (0.111)	0.221^*^ (0.120)	0.156^*^ (0.086)	0.608^**^ (0.267)
jiankang	−0.143 (0.205)	0.628^*^ (0.335)	0.069 (0.177)	0.000 (0.000)
ln_fincome1_per	0.473^***^ (0.146)	0.445^***^ (0.138)	0.476^***^ (0.110)	0.378 (0.265)
familysize22	−0.205^***^ (0.067)	−0.169^**^ (0.070)	−0.173^***^ (0.050)	−0.328^**^ (0.152)
1. Region	0.000 (0.000)	0.000 (0.000)	0.000 (0.000)	0.000 (0.000)
2. Region	−0.045 (0.242)	0.098 (0.247)	0.002 (0.183)	0.016 (0.572)
3. Region	0.030 (0.274)	0.125 (0.263)	−0.057 (0.206)	0.679 (0.483)
_cons	−1.507 (1.798)	−1.900 (1.973)	−0.948 (1.330)	−1.624 (3.308)
*N*	918	840	1,444	287
Pseudo *R*^2^	0.240	0.233	0.225	0.309
chi^2^	111.304	137.993	193.071	51.276

Models 1–4 perform regression analyses by stratifying the sample into female, male, rural, and urban groups, respectively, to conduct heterogeneity analysis.

Region 1–3 respectively refers to the eastern, central and western parts of China.

Robust standard error in parentheses; ^***^ p <0.01, ^**^ p <0.05, ^*^ p <0.1.

**Table 8:** Heterogeneity analysis of age and family per capita income.

**Variable**	**(5)**	**(6)**	**(7)**	**(8)**	**(9)**	**(10)**
	**Young**	**Middle-aged**	**Middle-aged and elderly**	**Low income**	**Middle income**	**High income**
factor1 (psychological and emotional value)	0.160^*^ (0.097)	0.231^*^ (0.128)	0.988 (0.799)	0.132 (0.170)	0.197 (0.150)	0.210^*^ (0.112)
factor2 (economic utility value)	0.266^**^ (0.112)	0.246^**^ (0.116)	−0.154 (0.587)	0.344^*^ (0.182)	−0.039 (0.142)	0.306^**^ (0.119)
factor3 (family responsibility value)	0.227^**^ (0.100)	0.324^***^ (0.123)	−0.108 (0.872)	0.273^*^ (0.163)	0.132 (0.147)	0.289^***^ (0.112)
Gender	−0.035 (0.200)	−0.653^***^ (0.240)	0.000 (0.000)	0.116 (0.287)	1.167^***^ (0.315)	0.497^**^ (0.239)
Age				−0.120^***^ (0.023)	−0.208^***^ (0.030)	−0.187^***^ (0.023)
hukou	0.768^**^ (0.302)	0.513 (0.313)	0.000 (0.000)	0.178 (0.525)	0.207 (0.378)	0.894^***^ (0.296)
medsure_dum	0.447 (0.389)	−0.543 (0.377)	−1.086 (1.670)	−0.081 (0.533)	−0.317 (0.483)	0.146 (0.480)
pensioninsurance_dum	0.142 (0.218)	0.007 (0.251)	−1.520 (1.163)	0.345 (0.304)	0.108 (0.286)	0.123 (0.285)
edu	0.257^**^ (0.110)	0.214^*^ (0.121)	−1.839^***^ (0.551)	0.077 (0.151)	0.251^*^ (0.153)	0.412^***^ (0.146)
jiankang	0.112 (0.233)	0.515 (0.357)	0.123 (0.580)	0.251 (0.326)	0.454 (0.434)	0.014 (0.229)
ln_fincome1_per	0.510^***^ (0.133)	0.427^***^ (0.158)	−0.155 (0.987)			
familysize22	−0.213^***^ (0.062)	−0.149^*^ (0.076)	−0.334^*^ (0.199)	−0.160^**^ (0.080)	−0.034 (0.082)	−0.450^***^ (0.096)
1. Region	0.000 (0.000)	0.000 (0.000)	0.000 (0.000)	0.000 (0.000)	0.000 (0.000)	0.000 (0.000)
2. Region	0.017 (0.229)	0.065 (0.279)	0.320 (1.340)	0.192 (0.357)	−0.034 (0.310)	−0.214 (0.267)
3. Region	−0.080 (0.249)	0.269 (0.284)	0.242 (1.224)	−0.189 (0.356)	−0.512 (0.398)	0.524 (0.321)
_cons	−7.103^***^ (1.687)	−7.574^***^ (2.088)	1.404 (8.560)	1.534 (1.455)	2.736 (1.667)	4.237^***^ (1.209)
*N*	565	875	210	601	571	586
Pseudo *R*^2^	0.117	0.081	0.243	0.116	0.238	0.295
chi^2^	64.682	39.248	56.896	45.561	65.462	117.804

Models 5–10 perform regression analyses by stratifying the sample into young, middle-aged, middle-aged and elderly, low-, middle-, and high-income groups, respectively (Models 5–7 for age stratification and Models 8–10 for income stratification), for the purpose of heterogeneity analysis.

The age group is divided by sex (female: 20–29/30–39/40–49; Male: 22–34/35–47/48–60).

Robust standard error in parentheses; ^***^p <0.01, ^**^p <0.05, ^*^p <0.1.

From the analysis of the heterogeneity in terms of gender and household registration, we found that the core independent variable, the psychological and emotional value (factor1), has a significant positive impact on females (0.316^**^) and the rural population (0.205^**^), but has a weaker influence on males (not significant) and the urban population (not significant). This reflects that the psychological and emotional drive is more important for the fertility intention of females and rural residents. The economic utility value (factor2) has a significant positive impact on females (0.255^**^) and the urban population (0.463^**^), and has a marginally significant impact on the rural population (0.157^*^), while it has no significant impact on males. This indicates that economic factors have a more prominent influence on the fertility decisions of females and urban residents, which may be related to the cost of living in cities or the economic status of females. The family responsibility value (factor3) has a significant positive impact on females (0.299^***^), the rural population (0.208^**^), and the urban population (0.488^**^), and has only a marginally significant impact on males (0.201^*^). This demonstrates that the family responsibility value is generally important, but has a stronger impact on females and the urban population (with a higher coefficient for the urban group). Among the control variables, it was found that household registration has a significant impact on the fertility intention of females (0.995^***^). The positive impact of the education level exists, but it has a stronger effect on the urban population (0.608^**^).

From the analysis of the heterogeneity in terms of age and income, it can be seen that the core independent variable, the psychological and emotional value (factor1), is significant for young people (0.160^*^) and middle-aged people (0.231^*^), but not significant for the middle-aged and elderly; it is marginally significant for high-income groups (0.210^*^). This reflects that psychological factors are more important for people of childbearing age (young/middle-aged) and high-income earners, and the fertility decisions of the middle-aged and elderly may be less driven by emotions. The economic utility value (factor2) is significant for young people (0.266^**^), middle-aged people (0.246^**^), and high-income groups (0.306^**^), and marginally significant for low-income groups (0.344^*^). However, it has a non-significant negative impact on the middle-aged and elderly and middle-income groups, indicating the confusion of the middle-aged, elderly, and middle-income groups about the economic value of children. The family responsibility value (factor3) has the strongest effect on middle-aged people (0.324^***^) and high-income groups (0.289^***^), followed by young people (0.227^**^). This reflects that family responsibility is particularly crucial for middle-aged people and high-income earners.

Key findings of the control variables: The gender coefficient in the middle-aged group is significantly negative (−0.653^***^), which may reflect that middle-aged men have a lower fertility intention, possibly because their childbearing needs have already been met. Household registration has a significant positive impact among young people and high-income groups (0.894^***^), suggesting that the combination of rural household registration with young people and high-income earners may strengthen the fertility intention. The education level has the greatest impact on high-income groups (0.412^***^), which may be because education enhances the emphasis on the quality of children. It has a significant positive impact among young and middle-aged people, and a significant negative impact among the middle-aged and elderly, suggesting that people of different age groups have different cognitive understandings of the concept of childbearing.

## 4 Discussion

This study, by constructing a multidimensional framework of children's value (encompassing psychological and emotional value, economic utility value, and family responsibility value), reveals the complex driving logic of fertility intention in the process of economic modernization. Our findings present three key revisions and advancements to mainstream fertility decision-making theories: (1) they challenge the single perspective of economic determinism and reveal the dominant role of the cultural dimension of family responsibility value; (2) they identify the promotive effect of educational attainment on the fertility intention of the new generation of reproductive-age groups, marking a paradigm shift from traditional demographic models; (3) they uncover the conditional impact of economic utility value that varies with resource endowments, and clarify the boundaries of economic rationality. The following sections will conduct an in-depth discussion around these three core findings.

### 4.1 The value of children is concentrated in three dimensions: psychological and emotional value, economic utility value, and family responsibility value

Hoffman and Hoffman (1973) divided the value of children into nine categories, which are the nine items in the value of children questionnaire, and these were later integrated into three to four dimensions. Through the value of children questionnaire, this study further confirms that the three major dimensions are psychological and emotional value, economic utility value, and family responsibility value. This represents the universality of the culture of the value of children across different cultures and its stability across different eras.

### 4.2 The three dimensions of the value of children all have significant positive impacts on fertility intention, and the family responsibility value has the greatest influence on fertility intention

Fertility decisions are jointly driven by utilitarian motives, emotional values, and family responsibilities, and the effects of these three factors are independent and complementary.

Among them, the psychological and emotional value indicates that the happiness experience of childbearing is a universal driving force that transcends economic conditions, confirming that emotional needs are an important source of resilience in the era of low fertility. The family responsibility value (with the strongest and most robust effect) confirms that traditional culture (such as “continuing the family line”) remains a deep anchor point for fertility decisions. Regarding the economic utility value, it is found that the effect is reversed after controlling for income and education, revealing its conditional promotion mechanism. That is, Economic-utilitarian values display conditional influences on fertility motivations. Low-income populations experience suppressed utilitarian demand due to prohibitive childrearing costs, whereas high-income groups reconstruct reproductive rationality through human capital investment frameworks. This dual-directional impact confirms the context-dependent nature of economic rationality's fertility effects, contingent upon individual resource endowment differentials. Females are more driven by psychological and emotional factors and family responsibilities, while for males, only family responsibility has a marginal impact. The rural population is influenced by psychological and emotional factors and family responsibilities, while the urban population relies more on economic and family responsibility values. Young/middle-aged people are jointly influenced by these three types of values, and high-income earners attach more importance to economic and family responsibilities.

### 4.3 The level of education has a positive effect on the fertility intention of the new generation group

Unlike most previous studies on fertility intention, which have generally pointed out a negative effect of educational attainment on fertility intention, this study draws a different conclusion: regardless of gender and household registration status, educational attainment exerts a promoting effect on the fertility intention of the new generation—an effect that is particularly evident among young people, middle-aged individuals, and high-income groups—and this significant positive effect may indicate an important paradigm shift.

This result may be attributed to the cultural specificity of the sample group. In terms of the logical chain, there is a positive transmission relationship of “education → fertility intention”. A common explanation is that groups with higher educational attainment often possess greater competitive advantages in the labor market due to their advantages in knowledge and skills, thereby enabling them to achieve higher income levels. Relatively abundant economic resources can effectively alleviate the cost pressures associated with childbearing, such as childcare expenses and educational expenditures, thus enhancing individuals' confidence and willingness to have children. Other explanations include the following: education may exert its influence through multiple pathways, such as shaping more egalitarian gender role perceptions, enhancing social capital for accessing childcare resources, and strengthening recognition of non-utilitarian values (e.g., the psychological and emotional value of children). The role of these aforementioned non-economic factors will be one of the most critical directions for future research.

It is worth noting that among the middle-aged and elderly groups, education has a negative effect on fertility intention. Evidently, the impact of education on fertility intention varies across different age groups. A possible reason is that the knowledge structure and cultural atmosphere in which the young generation is situated are significantly different from those of previous generations. They attach more importance to the family responsibility value of their children, such as the roles of children in family care for the elderly and emotional support. At the same time, they also value the economic utility value of their children, such as the potential economic returns and social status enhancement that children may bring in the future. The recognition of these two aspects of value has, to a certain extent, increased their motivation to have children. This provides a theoretical basis for implementing precise interventions segmented by age groups and social strata, and reveals the dynamic hierarchical logic existing in fertility decision-making.

## 5 The policy implications

Based on the findings of this study, we propose the following targeted policy recommendations. Firstly, public communication campaigns should leverage cultural resilience by reframing procreation as a societally valued endeavor rooted in cultural continuity, emphasizing intergenerational solidarity and the importance of family lineage, particularly targeting high-income and urban populations where family responsibility value is most influential. Secondly, economic incentives must be designed according to specific resource endowments; for low-income groups, direct financial support such as means-tested childbirth grants, housing subsidies linked to family size, and subsidized childcare is crucial to alleviate immediate cost barriers, while for middle- and high-income groups, incentives should focus on safeguarding human capital through significant tax credits for educational expenses and preferential admission policies for children from multi-child families into high-quality public schools. Finally, integrating fertility-positive education into existing systems is essential, including incorporating modules on family sociology and the non-material value of children into secondary school curricula, and strengthening workplace policies for highly educated professionals with flexible work arrangements, robust on-site childcare facilities, and explicit anti-bias protections for parents to reduce the opportunity cost of childbearing. These targeted measures, aligned with the distinct value structures and resource constraints of different subpopulations, offer a practical and effective pathway for addressing low fertility.

## 6 Limitations and prospects

Data Limitations: The content of the CFPS questionnaire is limited. In the future, cultural measurements (such as the strength of clan networks and the frequency of intergenerational interactions) can be added to enhance the ecological validity of value measurements.

Lack of Dynamic Mechanism: Cross-sectional data cannot capture the temporal evolution of fertility intention. It is necessary to combine longitudinal surveys to verify the life-cycle effects.

Endogeneity Problem: Cross-sectional data cannot completely rule out reverse causality (for example, people with a strong fertility intention may attach more importance to family responsibility).

## 7 Conclusion

This study goes beyond the single economic explanatory framework, develops a multidimensional value-driven model, and reveals the complex coupling mechanism between economic rationality and traditional culture in fertility decision-making. Its main theoretical contributions are reflected in three aspects: First, it identifies the dominant role of family responsibility value over economic motives, providing new evidence for understanding the cultural resilience mechanism under low fertility; Second, it uncovers the dual-directional impact of economic utility value that varies with resource endowments, clarifying the boundary conditions for the role of economic rationality; Third, it discovers the pro-natal effect of educational attainment among the new generation, challenging the traditional demographic paradigm. Collectively, these findings not only provide policy pathways that are both culturally sensitive and economically feasible for addressing the low fertility dilemma, but also contribute a perspective of Chinese experience to the theories of population sociology.

## Data Availability

Publicly available datasets were analyzed in this study. This data can be found here: http://www.isss.pku.edu.cn/cfps/sjzx/gksj/index.htm.

## Supplementary material

Table S1 Collinearity of independent variables.
