# Supplementary material for: The influence of the value of children on the fertility intentions of people of childbearing age in China
Source: Front Sociol. 2025 Nov 24;10:1686244. doi: 10.3389/fsoc.2025.1686244 (PMC12682769; doi:10.3389/fsoc.2025.1686244)
Supplement: Table S1 — Collinearity of independent variables. [file Data_Sheet_1.pdf]

Supplementary materials

Table S1. Collinearity of independent variables

```
.    reg qka205 factor1 factor2 factor3 gender age hukou spouse medsure_dum pensioninsurance_du edu    j
> iankang ///
>     ln_fincome1_per familysize22    ///
>    region , vce(robust)
note: spouse omitted because of collinearity.

Linear regression                               Number of obs     =      1,758
                                                F(13, 1744)       =      26.15
                                                Prob > F          =     0.0000
                                                R-squared         =     0.1860
                                                Root MSE          =     .33941
```

qka205	Coefficient	Robust std. err.	t	P>|t|	[95% conf. interval]	
factor1	.0187459	.0079941	2.34	0.019	.0030669	.0344249
factor2	.0300393	.0089035	3.37	0.001	.0125766	.047502
factor3	.0283783	.0083583	3.40	0.001	.011985	.0447716
gender	.052189	.0167491	3.12	0.002	.0193386	.0850394
age	-.0174677	.0012758	-13.69	0.000	-.0199701	-.0149654
hukou	.0753924	.0220192	3.42	0.001	.0322055	.1185793
spouse	0	(omitted)				
medsure_dum	-.0061776	.0330806	-0.19	0.852	-.0710594	.0587042
pensioninsurance_dum	.0248878	.0183152	1.36	0.174	-.0110343	.0608099
edu	.0185237	.0089664	2.07	0.039	.0009377	.0361097
jiankang	.0030472	.0124328	0.25	0.806	-.0213376	.0274319
ln_fincome1_per	.0554769	.0108856	5.10	0.000	.0341267	.0768271
familysize22	-.0247354	.0042801	-5.78	0.000	-.03313	-.0163407
region	.0085818	.0106297	0.81	0.420	-.0122665	.0294301
_cons	.1873199	.1406222	1.33	0.183	-.088486	.4631259

Variable	VIF	1/VIF
edu	1.73	0.579285
ln_fincome~r	1.46	0.684324
age	1.32	0.758711
hukou	1.21	0.823463
factor2	1.20	0.836542
familysize22	1.19	0.836843
pensioni~dum	1.14	0.873855
region	1.14	0.878752
medsure_dum	1.06	0.945592
gender	1.06	0.945807
jiankang	1.05	0.954220
factor3	1.03	0.967709
factor1	1.02	0.981619
Mean VIF	1.20	

.
